# Inflammatory signaling in NASH driven by hepatocyte mitochondrial dysfunctions

**DOI:** 10.1186/s12967-023-04627-0

**Published:** 2023-10-26

**Authors:** Melissa Myint, Francesca Oppedisano, Valeria De Giorgi, Byeong-Moo Kim, Francesco M. Marincola, Harvey J. Alter, Salvatore Nesci

**Affiliations:** 1 Sonata Therapeutics, Watertown, MA USA; 2 Department of Health Sciences, Institute of Research for Food Safety and Health, University “Magna Græcia” of Catanzaro, Catanzaro, Italy; 3 Department of Transfusion Medicine, Clinical Center, National Institutes of Health, Bethesda, USA; 4 Department of Veterinary Medical Sciences, University of Bologna, Ozzano Emilia, Italy

**Keywords:** NASH, Mitochondrial dysfunction, Ox-mtDNA, Inflammatory process, Hepatic stellate cells

## Abstract

Liver steatosis, inflammation, and variable degrees of fibrosis are the pathological manifestations of nonalcoholic steatohepatitis (NASH), an aggressive presentation of the most prevalent chronic liver disease in the Western world known as nonalcoholic fatty liver (NAFL). Mitochondrial hepatocyte dysfunction is a primary event that triggers inflammation, affecting Kupffer and hepatic stellate cell behaviour. Here, we consider the role of impaired mitochondrial function caused by lipotoxicity during oxidative stress in hepatocytes. Dysfunction in oxidative phosphorylation and mitochondrial ROS production cause the release of damage-associated molecular patterns from dying hepatocytes, leading to activation of innate immunity and trans-differentiation of hepatic stellate cells, thereby driving fibrosis in NASH.

## Introduction

Non-alcoholic fatty liver (NAFL) is the hepatic manifestation of metabolic syndrome, a collection of conditions that increase risk of coronary heart disease, diabetes, and stroke, amongst other serious health conditions. NALF in particular spans a spectrum of diseases from benign steatosis to non-alcoholic steatohepatitis (NASH), cirrhosis, and hepatocellular carcinoma [1]. The pathological characteristic of NAFL is hepatic steatosis, which is defined as an accumulation of fat, in the form of triglycerides, in more than 5% of hepatocytes mostly around the portal vein [2]. The accumulation of fat, caused by disruptions in fatty acid (FA) transport and metabolism, presents phenotypically as lipid droplet (LD) accumulation within hepatocytes. It is the cumulative result of de novo lipogenesis, adipose tissue dysfunction, and obesity-mediated insulin resistance associate to inflammation [3]. All of which impact FA oxidation, mitochondrial metabolism, and lipoprotein trafficking underlying metabolic-associated fatty liver disease (MAFLD) [4–8]. Overexpression of IGF2 in the liver can result in increased LD formation and free cholesterol accumulation, and may thus play a role in the start of steatosis [9].

According to a “*classical theory*”, NAFL develops through a first “hit” that triggers lipid accumulation and inflammation associated with insulin resistance and progresses through a second “hit” characterized by increased oxidative stress and lipid peroxidation, determining the progression from NAFL to NASH [4, 10]. Additional research refined the two-hit hypothesis into a multiple-hit hypothesis, which qualified that the accumulation of triglycerides actually sensitizes the liver to a multitude of secondary insults that include direct lipotoxicity and oxidative stress driven by free radicals from β- and ω-oxidation of free fatty acids (FFAs) [11]. The multiple-hit hypothesis also acknowledges contributions from increased intestinal permeability and alterations in the gut microbiota that result in endotoxin-driven inflammation through activation of Toll-like receptor-4 (TLR4) in Kupffer cells and hepatocytes [11]. Insulin resistance, changes in adipokine secretion, in addition to activation and senescence of hepatic stellate cells (HSCs) all ensue. The cumulative effect is inflammation of the liver and cellular damage, ultimately triggering fibrogenesis [11, 12].

**Fig. 1:**
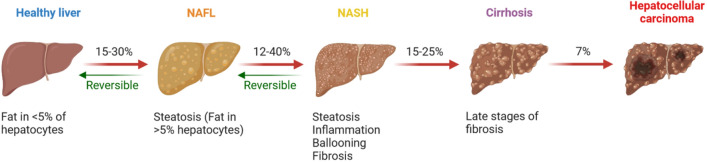
NAFL spectrum during development and progression. In healthy livers, fat accumulation in hepatocytes develops a non-alcoholic fatty liver (NAFL) that can progress from steatosis to a more severe form of steatohepatitis (NASH) characterized by inflammation and fibrosis. In most cases, but not all, NASH can be reversed to less severe disease through changes in lifestyle and diet. Continued fibrogenesis, however, drives progression of NASH to a state of irreversibility, eventually leading to cirrhosis and even hepatocellular carcinoma in some cases

Recent studies have shown a strong association between NAFL and the development of hepatocellular carcinoma even at the non-cirrhotic stage of disease (Fig. 1). The implication is that fatty liver and the associated inflammatory mediators may contribute trigger a pro-tumorigenic condition. Indeed, pro-inflammatory cytokines, such as TNFα or IL-6, contribute to the establishment of chronic inflammation in the adipose liver, enabling the progression to NASH and subsequently carcinoma. This is particularly evident in severely obese patients where levels of intra-hepatic IL-6 are notably high and are reduced with beneficial effects in subjects undergoing bariatric surgery [5, 13]. Moreover, inflammation can play a dominant role in deregulated signaling of the insulin/insulin-like growth factor system (IGFs) in obesity, diabetes, and cancer. In these inflammatory pathological conditions, the multiligand receptor for advanced glycation end-products (RAGEs) is driven by aberrant cross-communication with the impairment of insulin/IGFs in modulating the gene transcription and protein translation in cancer [14]. In addition, in NASH, the oxidative stress of biomolecules in the second hit event generates advanced glycation end products (AGEs), i.e. oxidated sugar reacting with lipids or proteins. AGEs promote the occurrence of NASH by RAGEs interaction through a cascade of downstream signaling triggering oxidative stress, hepatocyte ballooning, and HSCs activation. The effects of AGEs in aggravating NAFL are due to RAGE interaction [15, 16]. Patients with NAFL also suffer high prevalence of other malignancies and cardiovascular diseases suggesting that NAFL alone or in combination with other metabolic risk factors can drive extrahepatic disease. Many studies have been conducted to document associations between NAFL and chronic kidney disease, type 2 diabetes, cardiovascular disease, or colorectal cancer [17–19]. The involvement of other organs, such as adipose tissue, intestine, and muscle, defines NAFL as a systemic metabolic disorder [11, 12]. Regardless of any co-morbidities, individuals with NAFL suffer a clearly increased risk of end-stage liver disease, hepatocellular carcinoma, and mortality, compared to the age- and gender-matched general population.

## Mitochondrial rewiring in the pathogenesis of hepatic metabolic syndrome

NAFL is characterized by macro-vesicular fat accumulation in the hepatocytes. FA infiltration and lipid droplet formation activate β-oxidation in mitochondria, causing oxidative stress and mitochondrial dysfunction (MD) [20]. In particular, mitochondrial reactive oxygens species (ROS) initiate a vicious cycle of ROS-induced ROS production [21]. Damage of cellular biomolecules by mitochondrial ROS exacerbates MD and increases hepatocellular oxidative stress [22] and cellular damage [23].

Hepatic lipid metabolism is controlled by a combination of various FA turnover processes (i.e., FA absorption, synthesis, and elimination), influenced ultimately by the balance of de novo lipogenesis and β-oxidation. In MAFLD, the homeostasis between these pathways is disrupted, and hepatic lipid accumulation progresses together with chronic inflammation and fibrinogenesis [24]. Excessive lipid accumulation induces rewiring of hepatic metabolic programs that leads to the development of steatosis [25]. Exposure of hepatocytes to monounsaturated FAs results in lipid accumulation without changes in cell viability, whereas exposure to saturated FAs decreases cell viability, increases caspase activation and results in cell death [25]. Excessive levels of saturated FAs modify the composition of cardiolipin, phosphatidylcholine, and phosphatidylethanolamine present in the inner mitochondrial membrane (IMM) [26]. In particular dysfunction of cardiolipin, a phospholipid that maintains correct functioning of the IMM, alters the fluidity and exchange capacity of the membranes (Box 1).

Fat accumulation in the liver impairs FA oxidation, decreases mitochondrial ATP-synthesizing respiration, and reduces hepatic glucose production by gluconeogenesis. Biochemical changes induced by a high-fat diet cause an increase in calcium uptake, and abrupt increases in mitochondrial calcium concentrations are deleterious on mitochondrial ATP production [28, 29]. High levels of free calcium modulate the influx of sodium into the mitochondrial matrix through the sodium/calcium exchanger. Increased sodium levels interact with the phospholipids in the inner leaflet of the IMM [30], reducing their mobility and thereby decreasing IMM fluidity.

The respiratory chain (RC) of mitochondria transports H^+^ and transfers electrons from substrates to molecular oxygen, generating a protonmotive force to support the phosphorylation of adenosine diphosphate (ADP). The RC is composed of Complex I (CI, NADH-ubiquinone oxidoreductase), Complex II (CII, succinate dehydrogenase), Complex III (CIII, cytochrome *bc*_1_ oxidoreductase), and Complex IV (CIV, cytochrome *c* oxidase) as part of the mitochondrial electron transport chain. The connection between these enzyme complexes is maintained by two mobile electron carriers, coenzyme Q and cytochrome *c*. Coenzyme Q diffuses freely through the IMM between CII and CIII but not CI and CIII in the respirasome supercomplex (CI + CIII_2_ + CIV) (Box 2). In the latter, coenzyme Q undergoes reduction reactions on CI and oxidation when transferred to CIII. These transfers of coenzyme Q occur within the respirasome, preventing its complete mixing into the lipid phase of the IMM [31]. For this reason, the membrane fluidity does not influence the kinetic activity of the respirasome. On the contrary, the decrease in IMM fluidity harms coenzyme Q transfer from CII to CIII, which do not form supercomplexes (SCs), leading to ROS production with profound consequences for cellular metabolism [30].

The imbalance between saturated and unsaturated FAs decreases the proportion of linoleic and arachidonic acids in the phospholipids of mitochondria, causing a reduction in carnitine palmitoyltransferase I (CPT1) activity. As a consequence, defects ensue in the conversion of long-chain acyl-CoA species to the corresponding long-chain acyl-carnitines to be transported into the mitochondria, where β-oxidation takes place. Malonyl-CoA inhibits FAs association to carnitine by regulating the enzyme carnitine acyltransferase, thereby preventing them from entering the mitochondria, where their oxidation and degradation occur. In hepatic steatosis, the synthesis of the CPT1 inhibitor malonyl-CoA is promoted with stimulation of glycolysis [28] and contribution to liver lipotoxicity [26, 32].

Defective hepatic mitochondrial oxidative metabolism of FAs is compensated by FA oxidation in the peroxisomes through β-oxidation and, in the case of lipid overload associated with NAFL, ω-oxidation in the endoplasmic reticulum. These alternative pathways of lipid oxidation generate significant amounts of ROS, oxidative stress, and toxic dicarboxylic acids, which all promote inflammation [23, 33]. These effects could be secondary to malonyl-CoA accumulation inhibiting CPT1, which in turn inhibits mitochondrial β-oxidation [34].

Biochemical balance of two conflicting processes, lipid anabolism and catabolism, occurs in different mitochondrial populations within the same cell [35]. Cytoplasmic mitochondria (CM) and lipid droplet-associated mitochondria are distinct populations present in the liver [36]. In LD-associated mitochondria, the high oxygen consumption linked to mitochondrial respiration during FA oxidation is not supported by an increase in ATP production. Furthermore, mitochondrial respiration supporting ATP synthesis in LD-associated mitochondria is lower than it is in CM. Uncoupling proteins (UCPs) decouple oxidative phosphorylation (OXPHOS) with their proton leak activity, impairing ATP synthesis. However, decreased efficiency in ATP synthesis is not due to their action. Instead, in LD-associated mitochondria, SCs exhibit reduced enzymatic kinetics [37]. NADH and FADH_2_ oxidation depends on different pools of mobile, diffusible coenzyme Q channeled within the respirasome [31]. A low endogenous pool of coenzyme Q and super-assembled respiratory complexes are bioenergetic features attributed to less active LD-associated mitochondria relative to the more active CM [37]. Thus, the loss of the supramolecular organization of mitochondrial SCs and the generation of ROS impair the bioenergetic balance in NAFL [38], playing a key role in its development [28], and the transition from simple steatosis to NASH.

Recently, three mitochondrial subpopulations have been proposed in hepatocytes: endoplasmic reticulum (ER)-mitochondria, LD-associated mitochondria, and CM. ER- and LD-associated mitochondria are functionally distinct from the latter. This may explain disparities in mitochondrial oxidative activity in NAFL, since the oxidative function of each population can be fully autonomous within a single hepatocyte. FA oxidation and Krebs cycle respiration can be elevated simultaneously in NAFL because these competing metabolic pathways that typically cancel each other out within a single mitochondria coexist in hepatocytes by residing in distinct mitochondrial populations [39].

Under the pathologic conditions of NAFL, excess citrate is synthesized in ER- and LD-associated mitochondria and exported into the cytosol. Accumulation of cytosolic citrate drives the accumulation of malonyl-CoA, which in turn drives FA synthesis while inhibiting FA oxidation. Thus, both ER- and LD-associated mitochondria are mainly lipogenic. Conversely, CM are responsible for FA oxidation, ketone body production, ureagenesis, and gluconeogenesis [28, 35]. LD expansion in the liver promotes mitochondrial recruitment to LDs, and consequently, these mitochondria support lipogenesis while antagonizing lipolysis with malonyl-CoA production.

Perilipins are members of an evolutionarily conserved protein family that includes perilipin, adipophilin, and TIP47. These LD scaffold proteins coat the lipid storage droplets of cells [40] and play additional important functional roles via interactions with mitochondria. Perilipin 5 recruits mitochondria at the LD surface and contributes to FA accumulation, increased citrate synthesis, and decreased FA oxidation [41]. Thus, overexpression of perilipin 5 promotes LD formation and LD-associated mitochondria, reduces ROS levels, and upregulates the activity of lipogenic enzymes [42]. The net effect is the induction of steatosis without causing inflammation. Conversely, perilipin 5 deletion prevents hepatic steatosis by improving β-oxidation in hepatocytes but causes hepatic damage and inflammation due to lipotoxicity [43]. In summary, perilipin 5 presence is linked to several LD-associated mitochondrial activities; particularly in avoiding the mitochondrial FA oxidation, perilipin 5 prevents the uncompensated lipotoxicity responsible for impaired mitochondrial function, ROS production, and induction of inflammation. Thus, perilipin 5 can be a potential therapeutic target in NAFL [42].

LD-associated mitochondria can bear both beneficial and detrimental effects on systemic lipid metabolism. Understanding the LD-associated-to-cytoplasmic mitochondria ratio and the mechanisms governing it may help the understanding of mitochondrial dysfunction [35, 44]. In particular, lipid toxicity is prevented by opposite metabolic actions of LD-associated mitochondria and CM. The former produces ATP and citrate required for synthesis of FAs that will be stored in the LDs, whereas catabolic FAs oxidation in CM sustains production of keto bodies that are exported to extrahepatic tissues. By diverting the products of FA oxidation for synthesis of keto bodies, over using them to fueling OXPHOS, ROS production is decreased overall [44].

The development of metabolic imbalances in obesity may be influenced by excessive ER-mitochondrial coupling, a crucial aspect of organelle dysfunction. Indeed, marked reorganization of mitochondria-associated ER membranes leads to calcium overload in mitochondria, decreased mitochondrial oxidative capacity, and increased oxidative stress [29]. Calcium accumulation and ROS production are responsible for changes in mitochondrial permeability driven by the formation of permeability transition pores (PTP), resulting in regulated cell death (RCD) [45]. In NASH, damaged mitochondria accumulate due to sustained failure of repair mechanisms, defects in mitophagy, and/or the selective degradation of mitochondria by autophagy. All of which, in their normal state, serve as standard mechanisms of quality control that enable cells to remove damaged mitochondria. Thus, a higher cellular mitochondrial mass in NASH may be related to decreased degradation of damaged mitochondria rather than increased mitochondrial biogenesis [46].

Impaired mitophagy occurs early in the pathogenesis of NAFL, and loss of PARKIN, a protein involved in mitochondrial biogenesis, exacerbates the progression of NAFL through its involvement in mitophagy signaling on the outer mitochondrial membrane (OMM) of damaged mitochondria. Specifically, PARKIN facilitates ubiquitination of OMM proteins to mark injured mitochondria for autophagosomal degradation. This phenomenon depends upon PARKIN recruitment and PINK1-dependent phosphorylation of both PARKIN and OMM ubiquitin. Genetic deletion of PARKIN promotes liver injury and steatosis, and the presence of impaired mitochondria is exacerbated by both liver fat accumulation and insulin resistance in response to high-fat diet [47–49].

Glutathione and thioredoxin are low molecular weight, thiol-containing compounds that act as ROS scavengers in antioxidant systems and modulate ROS-associated damage. The redox potential of mitochondrial glutathione and thioredoxin is determined by NADPH reserves and the NAD(P)-transhydrogenase-dependent activity of nicotinamide nucleotide transhydrogenase (NNT), an enzyme that plays an important role in supporting redox homeostasis [50]. In NNT-null mice, diet-induced progression of steatosis to steatohepatitis is exacerbated, relative to wild-type, NNT^+^/^+^ mice [51]. Since NNT is a reversible enzyme exploiting the mitochondrial protonmotive force in the forward enzymatic reaction, NNT reduces NADP^+^ at the expense of NADH oxidation producing NADPH and NAD^+^. The missing activity of the enzyme decreases cell sensitivity to energy demand. As a consequence the Krebs cycle and β-oxidation activities decrease [52]. Moreover, sirtuin 3 (SIRT3), a mitochondrial NAD^+^-consuming enzyme, facilitates post-translational modifications (PTMs) of proteins required for normal cellular function. The SIRT3 catalytic activity is enhanced by NAD^+^ synthesized by NNT working in the forward reaction producing NAD^+^ and NADPH. SIRT3 displays robust PTM, i.e*.*, deac(et)ylase activity NAD^+^-dependent in mitochondria, acting positively on longevity and energy homeostasis [53].

High ratios of NAD^+^/NADH stimulate increased mitochondrial SIRT3 activity, which is beneficial to human health. The phenomenon is positively linked to the NNT-forward mode of catalysis [50]. Conversely, poor SIRT3 activity can correlate with the predisposition to certain diseases, among which NASH [54]. For example, in a study where mice were fed a high-fat or methionine-choline-deficient diet, increased susceptibility to diet-induced NASH correlated with reduced SIRT3 activity. The role of SIRT3 in the progression of NASH is related to its deacetylation PTM action on manganese superoxide dismutase (Mn-SOD or SOD2) and the Nucleotide-binding Oligomerization Domain (NOD)-like receptor family pyrin domain containing 3 (NLRP3) inflammasome. With the former, SIRT3 plays critical role in increasing the deacetylation of Mn-SOD, which ultimately decreases the anion superoxide levels in mitochondria, whereas with the latter, SIRT3 plays a role in blocking NLRP3 inflammasome activation, which ultimately suppresses certain inflammatory responses involved in NASH pathogenesis [55].

Box 1 Membrane fluidity The biological membrane can move lipid moieties within the lipid bilayer to enable functions like energy transfer, carrier-mediated transport, and regulation of enzyme activity. The free movement of lipid and/or protein constituents within the cell membrane is a parameter known as fluidity. Lower temperatures decrease the fluidity of membranes, whereas higher temperatures have the opposite effect. At temperatures in the physiological range, long-chain saturated fatty acids, e.g., palmitic acid (16:0) and stearic acid (18:0), tend to reduce the fluidity of membranes. Conversely, unsaturated fatty acids increase membrane fluidity [27].

Box 2 Superassembly of respiratory complexes Mitochondrial respiratory complexes I, III, and IV can be organized into SCs. SCs are formed by different compositions of respiratory complexes. Complexes I + III_2_, I + III_2_ + IV, and Complex III_2_ + IV are the most ubiquitous SCs detected. On the contrary, CII is found uncomplexed to other respiratory complexes to form SCs. CI and CII oxidize NADH and succinate, respectively, and coenzyme Q is reduced, shuttling the electrons from CI and CII to CIII. Then, cytochrome *c* exchanges electrons from CIII to CIV. Organization into SCs: CI + CIII_2_ + CIV (PDB ID 5J4Z), CI + CIII_2_ (PDB ID 6QBX), and CIII_2_ + CIV (PDB ID 7O3C) are drawn as ribbon representations obtained from modified Protein Data Bank IDs. The differently colored letters identify the complexes.
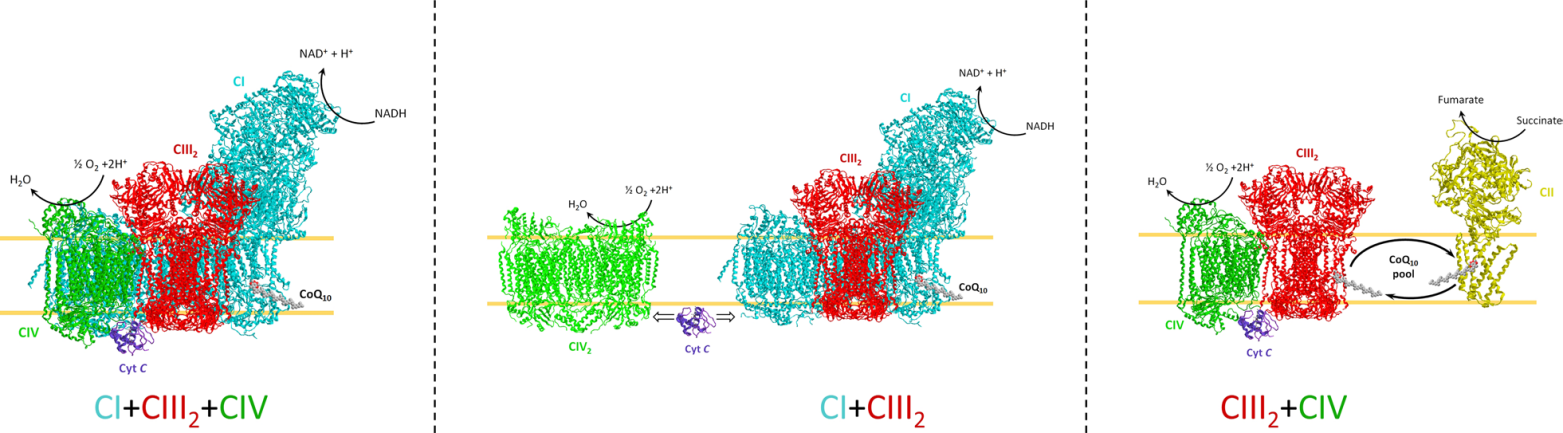

## The crosstalk between mitochondria and inflammation

NASH is caused by lipotoxicity and activation of the innate immune system. The “two-hit” concept previously discussed suggests that deposits of fat cause a “first hit” followed by a “second hit” of oxidative stress, lipid peroxidation, and necroinflammation that leads to NASH. However, the pathogenesis of NASH should also account for the combined effects of additional biochemical and immunological processes involved in complex interactions between hepatocytes and the immune system [34]. Inflammation arising in a state of over-nutrition and excessive lipid storage without infection is termed “*metabolic inflammation*” [56], where imbalances in mitochondrial ROS production cause oxidative stress and lipotoxicity, leading to mitochondrial DNA damage, lipid peroxidation, and the release of proinflammatory cytokines. Additionally, ATP depletion during MD is associated with inflammatory reactions that occur with certain forms of regulated cell death, such as apoptosis, necrosis, necroptosis, and pyroptosis [34, 57].

Kupffer cells, the resident macrophages of the liver, detect hepatic injury by recognizing danger-associated molecular patterns (DAMPs) or pathogen-associated molecular patterns (PAMPs). The presence of PAMPs, such as lipopolysaccharide (LPS) and flagellin, can occur in the liver from altered compositions of gut microbiota and/or increased intestinal permeability. DAMPs, on the other hand, are primarily derived from damaged hepatocytes and consist of ATP, uric acid, cholesterol crystals, FAs, and mitochondrial DNA (mtDNA), which can exist in the form of nucleoids or fragments of non-oxidized and oxidized mtDNA [58]. The latter, identified as ox-mtDNA, is the result of oxidative stress stemming from MD [59].

Mitochondria are recognized as key regulators of innate immunity, and it is now known that mtDNA released into the cytoplasm, outside the cell, or into the extra-cellular milieu activates several innate immune signaling pathways [60, 61]. Moreover, prokaryotic bidirectional transcription of mtDNA generates mitochondrial double-stranded RNA (mtds-RNA). In polynucleotide phosphorylase-depleted cells, mtds-RNA enters the cytosol through BAX–BAK mitochondrial pores, activating innate immune RNA sensors via type 1 interferon responses, such as those mediated by IRF1 [62].

**Fig. 2:**
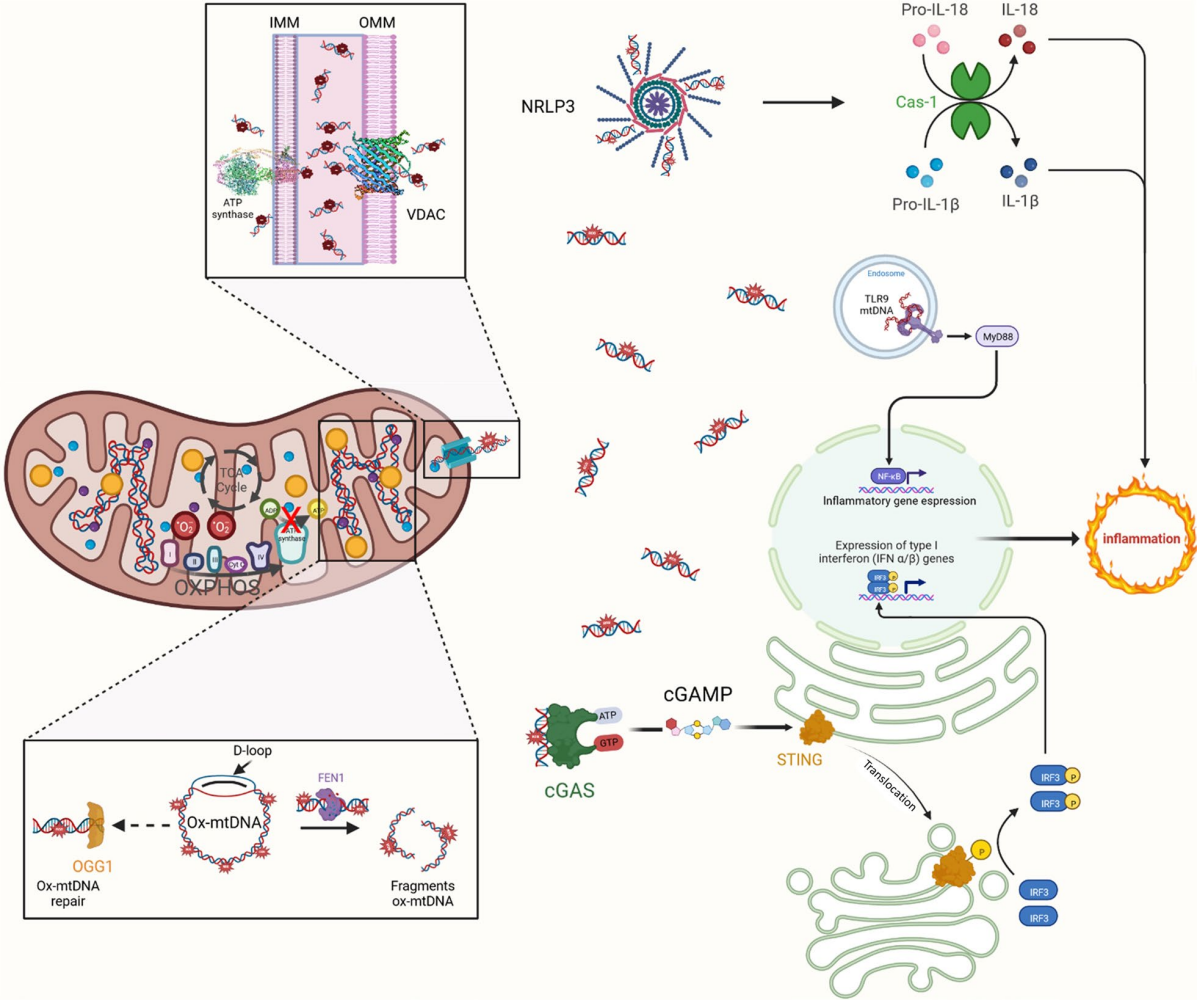
Liver inflammatory processes under conditions of oxidative stress in impaired mitochondria. Mitochondrial dysfunction triggers ROS production in OXPHOS. The damaged mtDNA formed under stress conditions is released into the cytosol, triggering certain inflammatory processes. NRLP3 inflammasome activation, TLR9 signaling, and activation of the cGAS-STING pathway generate inflammatory mediators, such as cytokines, including interferons, or IFNs. *OXPHOS* oxidative phosphorylation, *OGG1* 8-oxoguanine DNA glycosylase-1, *FEN1* Flap Structure-Specific Endonuclease 1, *VDAC* voltage-dependent anion channel, *IMM* *and* *OMM* inner and outer mitochondrial membrane, respectively, *NRLP3* NLR Family Pyrin Domain Containing 3, *IL* interleukin, *Cas-1* caspase 1, *TLR9* toll-like receptor 9, *MyD88* myeloid differentiation primary response 88, *cGAS* cyclic GMP–AMP synthase, *cGAMP* cyclic guanosine monophosphate–adenosine, *STING* stimulator of interferon genes, *IRF3* interferon regulatory factor 3. Figure created with BioRender (http://www.BioRender.com)

MtDNA release occurs through additional mechanisms, which can include IMM herniation followed by BAK–BAX pore formation in the OMM and/or defective mitophagy. In general, unoxidized nucleoids of mtDNA are released by BAK/BAX pores, whereas ox-mtDNA release is mediated through the PTP and voltage-dependent anion channel (VDAC) pores under oxidative stress [60] (Fig. 2). Ultimately, a wide range of stresses can cause the release of mtDNA, e.g., alteration of mtDNA homeostasis preventing replication, segregation, or damage, mitochondrial OXPHOS dysfunction increasing ROS production, inhibited electron transport, rewiring of cellular metabolism of cholesterol, fatty acids, or pyrimidines, impairment of mitophagy or autophagy, and/or ER stress.

MtDNA is a circular double-stranded molecule consisting of a heavy chain rich in guanine nucleotides, a light chain rich in cytosine, and a control region comprising the main non-coding region of the mitochondrial genome [63]. The non-coding region of the mtDNA molecule is also known as the displacement loop (D-loop), and it contains essential elements for DNA replication and transcription [64]. The D-loop is prone to oxidative damage, particularly in the form of 8-hydroxy-2-deoxyguanosine (8-oxoG). When the D-loop expands during mtDNA replication, an increase occurs in the formation of ox-mtDNA. Indeed, the most common type of DNA mutation arises from ROS-driven modifications of guanine (G), leading the formation 8-oxoG. Oxidized guanine can mismatch with adenine through formation of a Hoogsteen base pair, or a non-Watson–Crick base pair, which is a type of base pairing between nucleic acids that can result in a mismatched linking with adenine (A) in the genome and lead to C → A transversions [65].

A major repair mechanism of oxidative damage in mitochondria occurs through 8-OxoG Glycosylase 1 (OGG1). Impairment in OGG1 function results in elevated amounts of 8-oxoG in mtDNA [66]. Mitochondrial dysfunction can also lead to inefficient OGG1-mediated repair, contributing to elevated ox-mtDNA generation. The oxidated DNA becomes a target for cleavage by the Flap Endonuclease-1 (FEN1), which together with ox-mtDNA and ROS production produce mitochondrial DAMPs. FEN1 cleavage of oxidized DNA results in 500–650 bp ox-mtDNA fragments that can exit through the pores of the mitochondrial membranes and into the cell cytoplasm, creating inflammatory signals [67].

Furthermore, MD, under an overload of mitochondrial calcium and ROS, increases and sustains the formation of PTP. The bio-architecture of PTP [68] appears to be an adaptive spillage function of ox-mtDNA transport through the IMM. Once activated, ox-mtDNA is translocated into the cytosol by oligomerization and opening of VDAC [69] (Fig. 2). Mitochondrial pores through the IMM and the OMM by PTP and VDAC oligomerization, respectively, support mitochondrial depolarization, leading to exacerbated production of ox-mtDNA and its fragmentation [69].

Innate immune signaling by cytosolic ox-mtDNA or mtDNA triggers activation of the NLRP3 inflammasome and DNA-sensing cyclic GMP–AMP (cGAMP) synthase (cGAS), which in turn activate stimulator of interferon genes (STING). Each component triggers the secretion of proinflammatory cytokines [67, 70, 71]. Additionally, cytosolic mtDNA can trigger the endosomal Toll-like receptor 9 (TLR9), which activates NF-κB-dependent inflammatory signaling. TLR9 is primarily localized in the ER and translocates to the endosome after stimulation by hypomethylated CpG motifs found in bacteria and mtDNA released into the cytosol in conditions of MD. Dimerization of TLR9 is required for activation and occurs through the recognition of two TLR9 protomers binding CpG motifs from different DNA molecules [72]. In this conformation, the cytosolic domain of TLR9 promotes the activation of the myeloid differentiation primary response 88 (MyD88) pathway and the production of inflammatory cytokines [73]. TLR9 has been particularly well studied in liver pathologies, and high quantities of mtDNA capable of activating TLR9 have been discovered in the plasma of humans and mice with NASH [61, 74].

Mitochondrial stress, causing ROS production and ox-mtDNA release, is a crucial and likely the most ubiquitous characteristic of NLRP3 inflammasome activation under many different pathological conditions [75, 76]. Although it is known that ox-mtDNA is a primary activating signal for the NLRP3 inflammasome, NLRP3 itself lacks a DNA-binding domain. Therefore, it is unclear how ox-mtDNA interacts with NLRP3 directly and whether other DNA-binding components of the NLRP3 system exist (Fig. 2).

The NLRP3 inflammasome is a multi-protein complex that activates caspase 1 via an adapter molecule known as ASC, or apoptosis-associated speck-like protein containing a caspase recruitment domain (CARD). Once activated, caspase 1 then induces the production of mature IL-1β and IL-18 through cleavage of their respective precursors, pro-IL1β and pro-IL-18. Activated caspase 1 can also facilitate a type of programmed cell death known as pyroptosis, which is mediated through gasdermin D [77, 78]. Thus, the NLPR3 inflammasome is composed of a sensor (NLRP3), an adaptor (ASC), and an effector (caspase1). The sensor can be activated by a variety of signals of which two are required: (i) a priming signal that up-regulates the expression of inflammasome components and (ii) an activation signal that promotes oligomerization of the inflammasome components.

Excessive production of ROS can play a role in inflammasome activation through the increased production of ox-mtDNA [76]. Although a clear and unified mechanism has not been established, both intracellular and extracellular mtDNA participate in promoting NLRP3 inflammasome activation: intracellular mtDNA as a direct activating ligand for NLPR3 and extracellular mtDNA as a DAMP involved in the priming and activation of the inflammasome [78]. In the former scenario, ox-mtDNA directly associates with NLRP3 and the PYD domain of NLRP3 may then attract ASC via homozygous PYD-PYD binding. The carboxy-terminal CARD of ASC may subsequently combine with the CARD domain of caspase 1 and activate it. Caspase 1 self-cleavage results in the formation of the NLRP3-ASC-caspase 1 inflammasome complex [78].

Moreover, activation of cGAS is induced by the dimerization of enzymes on two fragments of mtDNA, and the 2cGAS:2mtDNA stoichiometry has each monomer of cGAS bound to two molecules of mtDNA [79]. Adenosine 5′-Triphosphate (ATP) and Guanosine 5′-Triphosphate (GTP) are converted by active cGAS into cyclic GMP-AMP, also known as 2′3′-cGAMP, which functions as a secondary messenger that stimulates STING on the ER membrane. Upon binding to cGAMP, STING polymerizes and translocates to the Golgi. Then, PTMs on STING stabilize the polymer, a step critical for subsequent interferon production. More specifically, active STING is phosphorylated, and this event triggers phosphorylation of interferon regulatory factor 3 (IRF3). IRF3 then dimerizes and translocates to the nucleus, where it stimulates the synthesis and secretion of type I interferons (IFNs) and other inflammatory cytokines [71]. Likewise, recent studies have indicated that the insulin/IGF axis may have a role in metabolic disorders, namely during the de-activation of aberrant IFN stimulation, contributing to the development of a successful strategy to prevent harmful IFN signaling [80].

FA metabolism can be a critical regulator of mtDNA-triggered cGAS-STING activation. Palmitic acid overload and lipotoxicity promote mtDNA release and cGAS-STING signaling [81], and mtDNA-STING-driven inflammation contributes to the pathophysiology of multiple high-fat diets [60, 82]. STING signaling triggered by the release of mtDNA is markedly activated in Kupffer cells after LPS treatment. LPS increases dynamin-related protein 1-dependent mitochondrial fission and, consequently, mitochondrial ROS generation, which causes mtDNA leakage into the cytosol and subsequent STING signaling activation [83, 84] (Fig. 2).

## Immunity and inflammatory process interplay in NASH

**Fig. 3:**
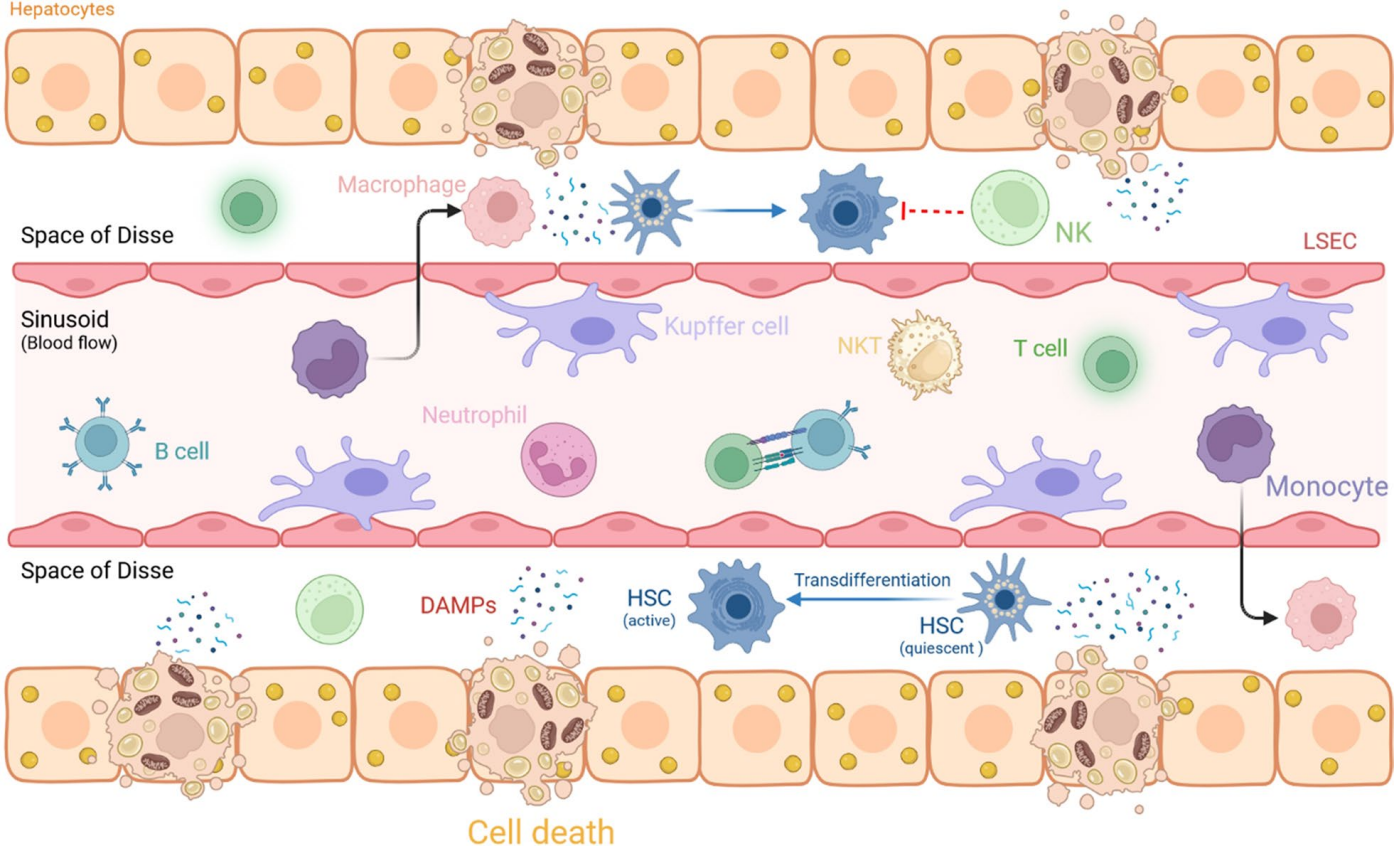
Crosstalk between non-parenchymal and surrounding immunological cells in NASH. *LSEC* liver sinusoidal endothelial cells, *NK* natural killer, *NKT* natural killer T, *HSC* hepatic stellate cell, *DAMPs* damage-associated molecular pattern. Figure created with BioRender (http://www.BioRender.com)

NASH is a disorder characterized by an active migration of immune cells into the liver (Fig. 3), where they undergo activation and acquire the ability to release mediators of inflammation. The resulting inflammatory process is associated with activation of innate immune signaling pathways stemming from oxidative stress, which drives ROS generation and causes mitochondrial alteration of OXPHOS and the generation of DAMPs by hepatocytes. Hepatocyte RCD, including apoptosis and lytic forms of hepatocellular death, such as necrosis, necroptosis, pyroptosis and ferroptosis [85], drives activation of Kupffer cells and other non-parenchymal cells, such as HSCs. In particular, the TLR9 receptor in Kupffer cells is directly activated by mtDNA released by hepatocytes, triggering an inflammatory cascade [86]. Associated chemokine release leads to hepatic accumulation of bone-marrow-derived, CCR2-expressing monocytes that massively expands the local monocyte-derived macrophage pool into sites of local inflammation [87]. CCR2 is highly expressed in infiltrated macrophages but not Kupffer cells. The relevance to NASH pathogenesis of TLR signaling and Kupffer cell activation was further validated utilizing TLR-deficient animal models; animals with Kupffer cells, that lack TLR9 or TLR4, became resistant to NASH [88, 89].

Accumulation of inflammatory cells is greater in NASH than in steatosis [90]. While the resolution of inflammation could restore liver homeostasis, often the inflammatory response exacerbates damage to stressed hepatocytes, resulting in a vicious cycle described as necroinflammation [91]. Indeed, the progression of inflammation in NAFL is rarely linear. This may explain why, when liver histology is assessed at a single time point, the dynamics of inflammation are weaker prognostic feature than fibrosis, which, in contrast, is a more static parameter.

Our understanding of the pathophysiology of inflammation in NAFL remains limited. An oversimplified definition of inflammation may not account for the dynamism of immune cells preventing the distinction between disease-promoting compared to inflammation-resolving mechanisms. Current pharmacological strategies addressing hepatic inflammation attempt to either: (1) inhibit the primary reaction to pro-inflammatory signals targeting the initial activation and recruitment of immune cells, or (2) focus on modulating the complex immune cell crosstalk involved in hepatic inflammation [91].

Hepatocytes in NAFL are not only targets of necroinflammation but also actively orchestrate and amplify immune responses. The vicious circle of inflammation results from lipotoxicity in hepatocytes and the Kupffer cell reaction to signals of stress or injury released by hepatocytes and/or other extra hepatic tissues. Kupffer cells then activate the inflammatory process and recruit monocyte-derived macrophages via the release of chemokines. Subsequent cGAS-STING and NLPR3 inflammasome signaling drive inflammatory macrophage activation as well as fibrogenic responses by HSC transdifferentiation [92, 93]. All of which contribute to further propagation of disease.

Liver macrophages are composed by Kupffer cells and monocyte-derived macrophages. Extra-cellular ox-mtDNA in NASH is a powerful inflammatory signal that activates these cells via the cGAS–STING pathway [94–96]. In response to these stimuli, macrophages secrete cytokines, chemokines, and other soluble factors that contribute to fibrosis. Unsurprisingly, STING depletion in macrophages has been shown to reduce the inflammatory responses and the severity of liver fibrosis. In chronic liver injury, metabolic and inflammatory pathways are co-regulated, and in all likelihood, cholesterol metabolism in macrophages is likely more closely related to IFN responses observed in NASH that reprogram lipid metabolism to alter the balance between lipid synthesis and scavenging, rather than to responses that facilitate a decrease in lipid pool sizes [97]. However, it is unclear whether immunometabolism dysfunction is a cause or a result of hepatic steatosis [98].

The NLRP3 inflammasome has emerged as a critical mediator of the steatosis-to-NASH transition through its influence on ubiquitous pro-inflammatory and pro-fibrogenic determinants. The NLRP3 inflammasome in hepatocytes leads to pyroptosis, which results in the secretion of hepatocyte derived NLRP3 inflammasome components and their internalization by HSCs. Once incorporated by HSCs, these particles accelerate and perpetuate inflammasome-driven pro-fibrogenic stress signals [99]. In particular, NLRP3 inflammasome assembly in HSCs contributes to their activation and thus directly induces fibrosis [100]. Activation of the NLPR3 inflammasome, however, is only one component of innate immune signaling capable of driving fibrosis. For example, signaling by the microRNA miR-155 via TLR4 in macrophages and hepatocytes has been linked to alcohol-induced steatohepatitis and fibrosis. More specifically, miR-155 leads to the expression of lipid metabolism genes as well as pro-inflammatory cytokines and chemokines [101].

Although innate immune pathways are considered the major contributor to inflammation in NASH, recent research suggests that adaptive immunity also plays a role in the progression of disease [102].

## Transition to NASH: hepatic inflammation of hepatic stellate cell-immune interactions

Non-parenchymal populations (sinusoidal endothelial cells, HSCs) and liver-resident immune cells (lymphocyte and macrophages) act as immune sentinels. Oxidative stress damages the liver and causes activation of Kupffer cells and HSCs, leading to liver fibrosis [103] due to the formation of an extracellular connective tissue matrix synthesized by activated HSCs (aHSCs). In a healthy liver, HSCs are perisinusoidal cells that exist in a state of quiescence, where they store retinoid-containing lipid droplets. In response to injury, HSCs activate and morph into a myofibroblast-like cell type. They proliferate and develop contractile, inflammatory, and chemotactic functions, while increasing the production of extracellular matrix [104]. aHSCs are the main source of liver myofibroblasts, and they represent the organ’s principal fibrogenic cell type [105].

DAMPs generated by damaged or dying hepatocytes and endothelial cells can promote HSC activation and fibrosis either directly or indirectly. However, a major contribution to HSC activation in NASH is also mediated by paracrine interactions with macrophages and other immune cells (Fig. 3). Soluble pre-fibrogenic and proliferative signals contribute to HSC-driven fibrosis [106], among them are TGF, PDGF, FGF2, MCP1, CCL3, CCL5, and ROS. Other soluble factors such as IL-1 and TNF induce fibrosis by promoting HSC survival via the NFκB pathway [106]. Furthermore, Sonic Hedgehog, a ligand of the Hedgehog-signaling pathway, is produced by steatotic hepatocytes and contributes to HSC activation, leading to NASH progression [107].

Metabolic dysregulation in HSCs may be a key component of hepatic fibrogenesis. During activation, HSCs experience increased levels of autophagy, which drives lysosomal activation and the subsequent cleavage and consumption of retinyl esters in HSC lipid droplets. Consequently, lipids and lipid droplets within HSCs are depleted in the process of generating energy to sustain HSC activation [108]. Even though the depletion of lipid droplets is a classical feature of HSC activation, the contribution of retinoids in HSC activation is not fully understood [106].

Epigenetic modifications can also contribute to metabolic dysregulation in HSCs. For example, HSC activation is impacted by DNA methylation of the promoter region of the Peroxisome Proliferator-Activated Receptor gamma (PPARγ) gene, a ligand-dependent transcription factor that regulates lipid metabolism, inflammation, and energy homeostasis in the liver and is a suppressor of HSC activation [109]. PPARγ is silenced via hypermethylation of its regulatory region in activated HSCs as fibrogenesis advances [110, 111]. Finally, HSCs can form “inflammatory” HSC clusters with strong immunological and secretory pathway activity, suggesting that HSC-to-HSC crosstalk may be a significant contributor to the development of NASH [93].

Natural killer T (NKT) cells in the liver produce substances that control inflammatory and fibrogenic responses. NKT cells are particularly abundant in the sinusoids of healthy livers, where they provide intravascular immune surveillance, but in steatosis, they are reduced in number. NKT cells actively promote fibrogenesis in NASH; CD1d-deficient mice that lack NKT cells are protected from NASH-related fibrosis, whereas treatment of primary HSCs with α-galactosyl-ceramide-activated NKT cells stimulates activation of HSCs to become myofibroblast-like [112]. NKT cells increase liver fibrosis by producing osteopontin and hedgehog ligands, both of which can directly activate HSCs [113]. However, direct NKT–HSC interactions are not well characterized. Conversely, NK cells, functionally and phenotypically distinct from NKT cells, act as innate immune effectors that resolve liver fibrosis through direct cytotoxicity against activated HSCs [114]. However, in NASH, NK cells have reduced capacity to kill HSCs under conditions of insulin resistance and increased TGFβ signaling [93].

The disruption of T lymphocyte signaling, or their depletion can be protective against fibrotic liver disease. The T-cell pathology enabled by certain NASH-associated metabolic stimuli (e.g*.*, short-chain FA acetate and extracellular ATP) results in auto-reactive CXCR6+ CD8+ T cells that exhibit cytotoxic activity against hepatocytes [115]. Interestingly, this cytotoxicity occurs through a mechanism dependent on the purinergic receptor (P2RX7) and is distinct from major histocompatibility (MHC)-class-I-dependent cytotoxicity. Additionally, HSCs can provide molecular signals to directly influence T-cell function in NASH [93]. One example includes the exploitation of their role as the key hepatic source of vitamin A, which upon release can amplify certain subsets of regulatory T cells. While their role is still not well understood, evidence suggests that they may play a role in enhancing metabolic inflammation [116]. Similarly, retinoid acid signaling from HSCs also supports B-cell survival and activation [93], which can, in turn, propagate additional HSC activation through a prominent innate-like signaling function that is characterized by the release of pro-inflammatory TNFα and other cytokines. However, the exact mechanisms by which B-cells lead to fibrotic liver disease have not been defined [117].

Taken together, trans-differentiation of quiescent HSCs, the body’s main reservoir of vitamin A, into proliferative, fibrogenic myofibroblast-like cells is the outcome of metabolic dysregulation in HSCs. This dysregulation ultimately leads to energy-dependent signaling pathways that influence the interaction of HSCs with surrounding inflammatory cells and other HSC-activating mediators. Importantly, while the cell-to-cell interactions discussed here are critical influencers of HSC activation and subsequent fibrogenesis, other cell-based interactions with HSCs not specifically mentioned in this review cannot be ruled out as additional key mediators in driving fibrogenic pathologies of NAFL and NASH.

## From current to emerging therapies: changing paradigm of NASH prevention and treatment

Although NAFL/NASH is a disease with a high worldwide incidence, no standard therapy yet exists (Table 1). Drugs treating associated metabolic disorders, such as hyperlipidemia, insulin resistance, and hyperglycemia are being empirically tested. Mostly, NASH patients are encouraged to undertake lifestyle changes with increased physical activity and dietary restrictions. Weight loss with or without dietary changes is generally effective; overweight/obese subjects with biopsy-proven NASH experienced histologically documented improvements with as little as 5% weight loss. In these patients, greater weight loss is associated with an improvement not only in steatosis but also in necrosis, inflammation, and fibrosis. Similar results are seen when weight loss is achieved with bariatric surgery. Unfortunately, not all NASH patients can or are willing to modify their lifestyle. For this reason, a pharmacological approach is desirable, and recently researchers have tried to better understand the molecular mechanisms underlying the accumulation of lipids, oxidative imbalance, and fibrosis in the liver to develop a therapy capable of reducing the onset of hepatic steatosis [6, 7, 118, 119].

**Table 1:** Current therapeutic treatments and emerging therapies to cure NASH

Current therapies	
Lifestyle changes	Increased physical activity
	Specified dietary restrictions
Insulin-sensitizing agents	Metformin
	Pioglitazone
Antioxidant agent	α-tocopherol (vitamin E)
Lipid-lowering drugs	Statins
Emerging therapies	
Anti-inflammatory and/or antifibrotic treatments	Resmetirom
	Lanifibranor
	Firsocostat
	Aramchol
	PNPLA3 gene silencing
	Semaglutide
	Tirzepatide
	FGF19/FGF21 analogues
	Inhibition of HSCs activation into matrix-producing myofibroblasts
Treatments to reduce the immune response	Chemokine receptor inhibitors
	Galectin-3 inhibitors
	Anti-platelet modulators

Among the drugs used to treat steatohepatitis are insulin-sensitizing agents. Among them, metformin failed to reproduce the histological improvements in NASH patients observed in preclinical, proof-of-concept studies. Another insulin-sensitizing agent used to treat NASH is pioglitazone. While this drug does reduce inflammation and resolve NASH, it is rarely used because of its strong association with congestive heart failure. A-tocopherol (vitamin E) is another drug that improves histological features in non-diabetic, NASH patients, but at high doses in long-term therapies, it also increases the prevalence of prostate cancer. Lipid-lowering drugs, such as statins, are also used to treat NASH [120–126].

Both the U.S. Food and Drug Administration and the European Medicines Agency stated that the approval of a drug against NASH must be linked to histological improvement and/or resolution of fibrosis, as documented by sequential liver biopsies. For this reason, researchers have prioritized the development of anti-inflammatory and/or antifibrotic drugs. However, both inflammation and fibrosis are endogenous defense mechanisms involved in restoring homeostasis. Therefore, any drugs developed must strike a balanced compromise between the benefits obtained from reducing NASH against the risks of reducing these endogenous defense mechanisms.

While some drugs with limited efficacy have been approved, many more are under clinical evaluation with positive results in phase III clinical trials, such a Resmetirom, a thyroid hormone receptor-β agonist; Lanifibranor, a pan-PPAR agonist; Firsocostat, an acetyl-CoA carboxylase inhibitor; and Aramchol, a lipotoxicity modulator that reduces inflammation. Furthermore, inflammation can be reduced by modifying genetic variants through, for example, PNPLA3 gene silencing. Semaglutide, tirzepatide, and FGF19/FGF21 analogues are hormonal agonists that, together with microbiota and lifestyle changes, reduce extrahepatic inflammation triggered by signals from the gut, circulation, and adipose tissue.

Since stress and death of hepatocytes trigger both innate and adaptive immune responses, therapies utilizing chemokine receptor inhibitors, galectin-3 inhibitors, or anti-platelet modulators are also under evaluation to reduce the activation, recruitment, and responses by immune cells.

Moreover, strategies to directly inhibit fibrogenesis are being pursued to prevent activation of HSCs. These approaches may target integrins and cytokines, which antagonize soluble factors involved in activating HSCs via cellular crosstalk with immune cells or may be related to agonising nuclear receptor signaling.

Finally, cell-based therapies with restorative macrophages or engineered T cells with chimeric antigen-receptors can be pursued in cases of advanced fibrotic disease to deactivate or kill HSCs or degrade the matrix to facilitate the restoration to normal homeostasis [91, 127].

In conclusion, the complex interplay between inflammation and mitochondrial dysfunction represents a pivotal axis in the pathogenesis of NAFL/NASH. This review has discussed the elaborate roles of various inflammatory mediators and the impact of dysfunctional mitochondrial processes in driving the progression from simple steatosis to the more severe stages of NASH. A comprehensive understanding of these interconnected mechanisms not only expands our insights into disease etiology but also opens new paths forward for targeted therapeutic interventions that hold the potential of ameliorating NASH’s impact on global health. Future research endeavors and therapeutic developments will undoubtedly benefit from the foundation established by this comprehensive exploration.

## Data Availability

Not applicable.

## Abbreviations

cGAS: Cyclic GMP–AMP synthase
CM: Cytoplasmic mitochondria
CPT1: Carnitine palmitoyl transferase I
DAMPs: Danger-Associated Molecular Patterns
FAs: Fatty acids
FDA: Food and Drug Administration
FEN1: Flap endonuclease-1
HSCs: Hepatic stellate cells
IGFs: Insulin-like growth factor system
IMM: Inner MITOCHONDRIAL membrane
LD: Lipid droplets
LPS: Lipopolysaccharide
MAFLD: Metabolic-Associated Fatty Liver Disease
MD: Mitochondrial dysfunction
NAFL: Non alcoholic fatty liver
NASH: Non alcoholic steatohepatitis
NKT: Natural killer T
NLRP3: Nucleotide-binding Oligomerization Domain-like receptor family pyrin domain containing 3
NNT: Nicotinamide nucleotide transhydrogenase
OGG1: 8-OxoG Glycosylase 1
OMM: Outer mitochondrial membrane
OXPHOS: Oxidative phosphorylation
PAMPs: Pathogen-Associated Molecular Patterns
PTM: Post-translational modification
PTP: Permeability transition pore
RAGEs: Receptor for advanced glycation end-products
SC: Supercomplex
SIRT3: Sirtuin 3
STING: Stimulator interferon genes
TLR: Toll-like receptor
VDAC: Voltage-Dependent Anion Channel
